# Emerging biomarkers for non-invasive diagnosis and treatment of cancer: a systematic review

**DOI:** 10.3389/fonc.2024.1405267

**Published:** 2024-07-26

**Authors:** Suleiman Zakari, Nguedia K. Niels, Grace V. Olagunju, Precious C. Nnaji, Oluwabusayo Ogunniyi, Mercy Tebamifor, Emmanuel N. Israel, Sunday E. Atawodi, Olubanke Olujoke Ogunlana

**Affiliations:** ^1^ Department of Biochemistry, College of Science and Technology, Covenant University, Ota, Ogun State, Nigeria; ^2^ Covenant Applied Informatics and Communication - Africa Centre of Excellence (CApIC-ACE), Covenant University, Ota, Ogun State, Nigeria; ^3^ Department of Biochemistry, College of Medicine, Federal University of Health Sciences Otukpo, Otukpo, Benue State, Nigeria; ^4^ Biotechnology Centre, University of Yaounde I, Yaounde, Cameroon; ^5^ Department of Molecular Biology, New Mexico State University, Las Cruces, NM, United States; ^6^ Department of Biochemistry, Federal University Lokoja, Lokoja, Kogi State, Nigeria

**Keywords:** biomarkers, cancer diagnosis, non-invasive biomarkers, cancer treatment, biomarker sensitivity, specificity, or clinical utility

## Abstract

Cancer remains a global health challenge, necessitating continuous advancements in diagnostic and treatment strategies. This review focuses on the utility of non-invasive biomarkers in cancer diagnosis and treatment, their role in early detection, disease monitoring, and personalized therapeutic interventions. Through a systematic review of the literature, we identified 45 relevant studies that highlight the potential of these biomarkers across various cancer types, such as breast, prostate, lung, and colorectal cancers. The non-invasive biomarkers discussed include liquid biopsies, epigenetic markers, non-coding RNAs, exosomal cargo, and metabolites. Notably, liquid biopsies, particularly those based on circulating tumour DNA (ctDNA), have emerged as the most promising method for early, non-invasive cancer detection due to their ability to provide comprehensive genetic and epigenetic information from easily accessible blood samples. This review demonstrates how non-invasive biomarkers can facilitate early cancer detection, accurate subtyping, and tailored treatment strategies, thereby improving patient outcomes. It underscores the transformative potential of non-invasive biomarkers in oncology, highlighting their application for enhancing early detection, survival rates, and treatment precision in cancer care.

**Systematic review registration:**

https://www.crd.york.ac.uk/prospero/display_record.php?ID=CRD42023474749 PROSPERO, identifier CRD42023474749.

## Introduction

Cancer, a complex and multifaceted group of diseases, remains one of the most significant public health challenges worldwide (1, 2). Cancer remains a leading cause of morbidity and mortality worldwide, with an estimated 19.3 million new cases and 10 million cancer-related deaths in 2020 alone (3). The prevalence of various cancer types varies significantly, with breast, lung, colorectal, and prostate cancers being among the most common (4). Specifically, breast cancer accounted for 11.7% of new cases, while lung cancer was responsible for the highest number of cancer deaths at 18% (3, 5).

The development and implementation of biomarkers in cancer diagnosis and treatment have gained substantial traction in recent years (6). Biomarkers are biological molecules found in blood, other body fluids, or tissues, signaling an abnormal process, condition, or disease. They hold promise for early cancer detection, prognosis, and monitoring treatment response, thereby enhancing precision medicine. The quest for early diagnosis and effective treatment strategies is an enduring pursuit in oncology (6). While traditional approaches such as tissue biopsies have long served as cornerstones of cancer diagnosis and management, the emergence of non-invasive biomarkers is and revolutionizing the field. Non-invasive biomarkers encompass various molecules and analytes, ranging from circulating tumor DNA (ctDNA) and exosomes to microRNAs and metabolites (7, 8). These biomarkers promise early detection, real-time monitoring, and personalised treatment strategies. In an era of precision medicine, identifying and validating these biomarkers hold immense potential to transform the landscape of cancer care (9).

Significant progress has been made in the clinical trials for biomarker-based treatments. As of 2023, numerous clinical trials are actively investigating the efficacy of biomarkers in various cancers. For instance, trials for non-small cell lung cancer (NSCLC) focus on biomarkers like Programmed Death-Ligand 1 (PD-L1) and Epidermal Growth Factor Receptor (EGFR) mutations, with promising results leading to the approval of several targeted therapies (10). In breast cancer, HER2 and BRCA mutations are pivotal in guiding treatment decisions, with ongoing trials exploring new biomarker targets (11). Variations in the Androgen receptor and gene mutations of its coactivators have been studied extensively for various applications (12, 13). The utilization of non-invasive biomarkers is particularly noteworthy. Techniques such as liquid biopsies, which analyze biomarkers in body fluids like blood, urine, and saliva, offer a less invasive alternative to traditional tissue biopsies. This approach is beneficial for continuously monitoring disease progression and response to treatment, providing a dynamic view of the cancer’s evolution.

This review seeks to explore emerging biomarkers for non-invasive diagnosis and treatment of cancer. It delves into the evolving realm of non-invasive diagnostics, seeking to understand the latest trends, innovations, and future prospects. By scrutinizing the scientific literature and research developments, we aim to shed light on the groundbreaking potential of non-invasive biomarkers in the battle against cancer. Furthermore, we navigate through the intricacies of liquid biopsies, epigenetic markers, non-coding RNAs, exosomal cargo, and metabolites. Through a systematic lens, we embarked on an exercise to discern the critical role these emerging biomarkers play in advancing early detection, tailored therapies, and improved patient outcomes. It is hoped that this review will uncover the latest discoveries, innovations, and relentless efforts at improving the lives of those impacted by this unrelenting disease and consider the future possibilities in the ever-evolving field of oncology and cancer patient care.

## Methods

### Protocol and registration

The protocol was registered with the PROSPERO under the identification number CRD42023474749 (14). This research does not involve using humans or animals, so no institutional review board or ethics committee approval was deemed necessary for this review.

### Literature search strategy

The search for relevant studies was conducted in multiple databases, including PubMed, Scopus, Web of Science, and Google Scholar. Keywords and search terms employed included “non-invasive biomarkers,” “cancer diagnosis,” “cancer treatment,” and variations thereof. Medical Subject Headings (MeSH) terms and Boolean operators were utilized to refine search results. The search encompassed articles published up to 2023. No language restrictions were applied, and non-English articles were considered. Grey literature sources, such as conference proceedings and preprint archives, were explored for potentially relevant studies. Manual searches included examining the reference lists of key articles and contacting experts in the field for additional studies.

### Inclusion and exclusion criteria

Articles were included if they met the Population, Intervention, Comparators, Outcomes, and Study Design (PICOS) Criteria and provided relevant information on non-invasive biomarkers in cancer. Exclusion criteria included studies that did not pertain to cancer lacked full-text availability or were based on animal or *in vitro* models. No significant deviations from the PICOS framework were applied, ensuring a focused and comprehensive selection process. The PICOS framework employed to define inclusion and exclusion criteria are as follows:

- Population: Studies involving human subjects with a confirmed or suspected cancer diagnosis.
- Intervention: Articles investigating non-invasive biomarkers for cancer diagnosis and treatment.
- Comparators: Studies comparing the effectiveness of different biomarkers or approaches.
- Outcomes: Articles reporting outcomes related to biomarker sensitivity, specificity, or clinical utility.
- Study Design: Original research articles, systematic reviews, and meta-analyses.

### Study selection

**Figure 1:**
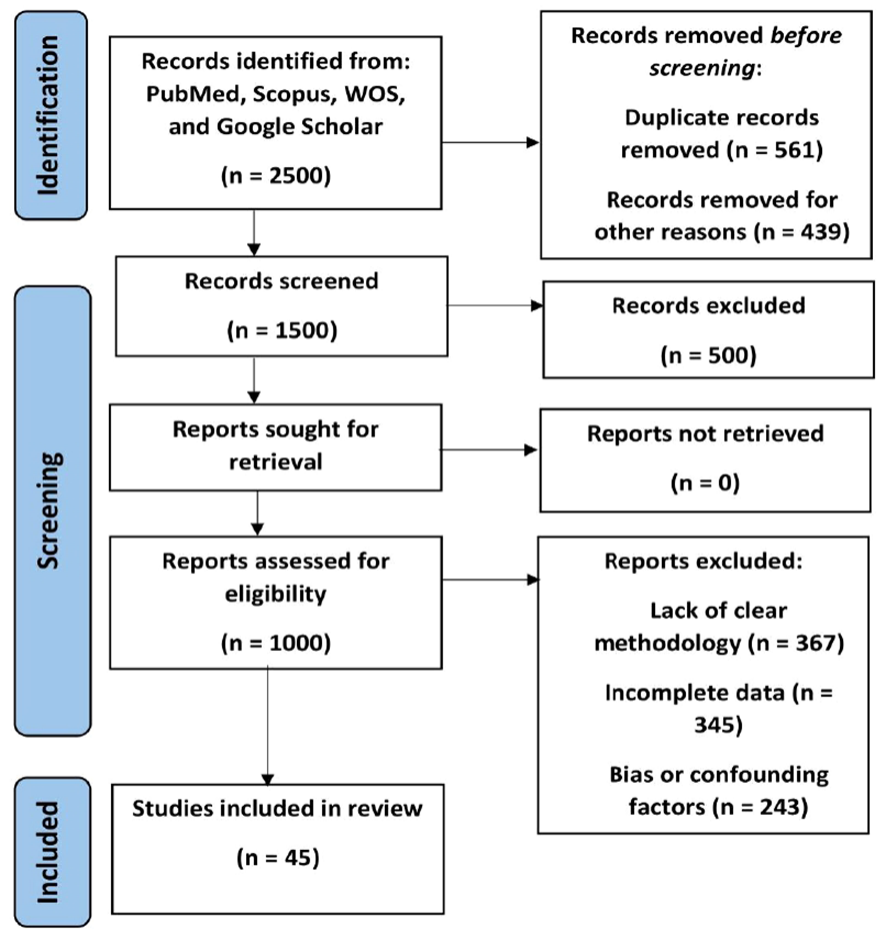
Study flow chart showing search results.

The selection process involved the following steps: Initial screening of titles and abstracts to identify potentially relevant articles. This was followed by a full-text review of selected articles to assess eligibility based on inclusion and exclusion criteria. Discrepancies between reviewers during title and abstract screening were resolved through consensus, and any remaining disagreements during full-text review were addressed through discussion. A flow diagram (**Figure 1**) illustrates the study selection process.

### Data extraction

The authors conducted individual literature reviews and documented their discoveries. Data extraction was conducted independently by two reviewers (SZ and NKN), and disagreements were resolved through discussion and consensus. To facilitate information extraction, a standard table containing characteristics of the studies included in the systematic study analysis was created. To mitigate selection bias, the authors cross-referenced their extracted data after the revision stage and addressed any discrepancies, while duplicate entries were eliminated. If disagreements persisted, a senior researcher (OOO) re-examined the data extraction process. Extracted data items included study characteristics (author, publication year), study design, population characteristics, biomarker types, outcomes, and key findings.

## Results

### Study selection

A search across various databases, including PubMed, Scopus, Google Scholar, and Web of Science, yielded 2,500 records. After an initial screening of titles and abstracts, 1,500 records were excluded due to irrelevance to the topic. The remaining 1,000 records underwent full-text assessment. After this thorough review, 925 articles were excluded for reasons including insufficient relevance, not meeting the inclusion criteria, and lack of full-text availability. After a meticulous screening process based on predefined inclusion and exclusion criteria, a further 30 articles were excluded for lack of relevance or inadequate methodology. Ultimately, 45 studies were included in the systematic review.

### Characteristics of included studies

**Table 1** presents a list of selected biomarkers categorized by cancer type, stage, and classification, as identified in the reviewed studies. The included studies exhibited diverse characteristics:

- Study Design: The selected studies encompassed a variety of designs, including cohort studies, case-control studies, randomized controlled trials, and systematic reviews.
- Population: These studies investigated populations with confirmed or suspected cancer diagnoses, covering a wide spectrum of cancer types.
- Interventions: The primary focus of the studies was on developing and evaluating non-invasive biomarkers for cancer diagnosis and treatment.
- Outcomes: The studies reported outcomes related to biomarker sensitivity, specificity, clinical utility, and their potential impact on cancer management.

**Table 1:** Characteristics of the studies included in the systematic study analysis.

Study I.D	Cancer type	Cancer stage	Biomarker	Classification	References
1	Prostate	locally recurrent CRPC	Bromodomain-containing proteins (BRDs)	Prognostic and prediction of treatment response	(1, 2)
2	Prostate	I	SPOP	Prognostic and disease characterisation	(3)
3	Colorectal	I-III	CTCF	Prognosis	(4)
4	Breast	I-III	AGAP2-AS1	Prognostic	(5)
5	Lungs	I	ALK, ROS-1	Predictive	(6, 7)
6	Breast	I-III	microRNA-1246	Diagnostic	(8)
7	NSCLC	I, II-IV	K-ras, p16^INK4A<^	Diagnostics	(9)
8	Prostate	I-III	ERG, PCA3, and SPDEF	Predictive and prognostic	(10)
9	Osteosarcoma	II-III	HSATI, HSATII, LINE1-P1, and Charlie 3	Predictive and prognostic	(11)
10	osteosarcoma	I-III	miR-92a-3p, miR-130a-3p, miR-195–3 p, miR-335–5 p, let-7i-3p	Predictive, diagnostic and prognostic	(12)
11	Prostate	I	TM256, KRAS	Diagnostic	(13)
12	Pancreatic	I, II-IV	glypican-1	Predictive and a	(14)
13	Lung	Early stage	Metabolome	Diagnostic, predictive and prognostic	(15)
14	Lung	Early stage	Circulating Tumor Cell	Diagnostic	(16)
15	Breast	Early and late stage	Metabolome	Prognostic and predictive	(17)
16	Prostate	T1	SPOP	Prognostic and disease characterization	(3)
17	Bladder	High-grade	Metabolome	Prognostic	(18, 19)
18	Prostrate	–	Free amino acids	Diagnostic	(20)
19	Cervical	Stage I,II & III	Metabolome and transcriptome	Diagnostic	(21–23)
20	All types	–	Circular RNAs (circRNAs)	Diagnostic and prognostic	(24, 25)
21	Breast Cancer	Stage I,II & III	microRNA	Diagnostic	(26)
22	Colorectal	adenocarcinoma stage	Microbial (microbiota) biomarkers	Prognostic and predictive	(18, 19)
23	Oral	Early stage	microRNA	Diagnostic and predictive	(27, 28)

CRPC; castration-resistant prostate cancer, NSCLC, SPOP; Speckle type POZ protein, CTCF; and others.

### Liquid biopsy biomarkers for arly cancer detection

Early cancer detection is the key to improved quality of life and survival and to reducing the financial burden of cancer treatments, which are greater at later stage detection (29). Liquid biopsy (LB) is a term used to describe the analysis of body fluid such as saliva, blood, urine, and cerebrospinal fluid, to identify specific biomarkers associated with cancer development and progression, for example, identification of Circulating Tumor Cells (CTC) as well as circulating tumor cells DNA in blood (29). In the context of cancer management, especially diagnosis and treatment, liquid biopsy which is the analysis of body fluids for circulating tumors cells, cell free nucleic acids, proteins or any other tumor fragments has opened new direction for early tumor detection and precision medicine (20, 21).

### Liquid biopsy and its significance in cancer diagnosis

The first liquid biopsy application in cancer analysis was circulating tumor cell detection which has different biomarkers depending on the type of cancer. Since many cancers are from epithelial cells there is a universal biomarker used for CTC detection epithelial cell adhesion molecules (EpCAM proteins). Its expression differs from one cancer type to another. It’s mostly used for breast and prostate cancer diagnosis (29). Liquid biopsy offers a non-invasive means for multiple clinical applications in cancer management including early cancer detection, staging and monitoring of localized cancer, predicting relapse and metastatic progression, assessing therapy efficacy, distinguishing early responders from non-responders, and tracking tumor evolution and resistance mechanisms, all of which can significantly enhance patient care and treatment outcomes (30). Therefore, liquid biopsies have a numerous advantages over the traditional biopsy (**Table 2**) as they help to obtain information from diagnosis to molecular profiling and response assessment without the need of tissue biopsy (31).

**Table 2:** Liquid biopsies advantages over traditional tissue biopsies.

	Advantages	References
Liquid Biopsy	It provides a clear understanding of the malignancies and tumorigenic biology from the blood, which is easy to get.	(31)
	No risk, non-invasive, painless	(8)
	Lead to personalize cancer management as possible for each patient	
	Lower procedural costs	(29)
	Easily repeatable	
	More reliable	
	Accessible for use in low- and middle-income countries	
	They are not contaminated with the use of preservatives like Formalin, freeze, paraffin	
	Provide fresh sources of reliable tumor’s derived components and materials	
	The analysis is rapid	
	Provide genomic, proteomic and metabolomic information	
Tissue biopsies	The gold standard in cancer diagnosis	(29)
	Help to determine the specific type of cancer	
	Enable more precise resection	

While liquid biopsies offer several advantages in cancer management compared to tissue biopsies, they are currently considered a secondary option in clinical applications. This is due to certain limitations (**Table 3**) that have hindered their full approval as the ‘gold standard’ for cancer diagnosis in clinical settings.

**Table 3:** Liquid biopsies limitation over traditional tissue biopsies.

	Limitations	References
Liquid Biopsy	Liquid biopsies are not considered as standard method for the diagnosis of cancer	(29)
	Liquid biopsies are used as complementary test to tissues biopsy	
	Liquid biopsy is less sensitive and specific than tissue biopsy	
	Liquid biopsy can lead to increased false positives and negatives	
	Liquid biopsy lacks the required accuracy in predicting tumor origin in cancer-positive patients	
Tissue biopsies	Invasive	(29)
	The process of analysis and getting the results are too stressful and long for the patient due to surgery	
	Tissue biopsies consist of an important risk of complications after surgery	
	Inability to capture tumor heterogeneity and its clonal tissue	
	Tissue biopsies are contaminated with preservatives like formalin, paraffin	

### Liquid biopsy biomarkers for cancer detection

Blood, cerebrospinal fluid (CSF), bone marrow (BM), saliva, sputum, cyst fluid, urine, and saliva are biological fluids that are relevant for liquid biopsy. These fluids can be analyzed to determine whether circulating tumor cells (CTCs), circulating tumor DNA (ctDNA), and other cancer-related biomarkers are present (30). Circulating tumor cells (CTCs) in peripheral blood are one of the emergent topics in cancer research since they can be used as a “liquid biopsy” technique. Noninvasive biomarkers have a clinical potential in the management of solid malignancies, such as prostate, ovarian, and breast cancer (7), for instance fibronectin (FN) which is present on the surface of extra vesicles released from human breast cancer cell lines, was considered as a potential biomarker candidate (32). This liquid biopsy technique to find FN on circulating EVs shows promise as a means of identifying specific markers for early breast cancer diagnosis. Thus, microRNAs enclosed in exosomes that are circulating in biofluids are an intriguing prospective biomarker for cancer because of their expression characteristics specific to cancer (22). In fact, Zhai LY et al. (22) reports an *in situ* detection of microRNA-1246 (miR-1246) in human plasma exosomes as breast cancer biomarker by a nucleic acid functionalized Au nanoflare probe (22). Recent investigations have shown that genetic alterations or epigenetic modifications in ctDNA could be used for cancer detection with a liquid biopsy like blood (33). Review of the existing literature reveals that methylation biomarkers play a critical role for the diagnosis and prognosis of certain malignancies. Additionally, certain publications describe a “PanCancer” a panel cancer detection technique for the simultaneous identification of multiple cancer types, demonstrating the promise of DNA methylation-based biomarkers for cancer detection and management (23). The use of liquid biopsies for cancer management has become more popular in clinical research and has many uses (24), including diagnosis, treatment and therapeutic monitoring. Given that DNA methylation in plasma can be identified early in the development of cancer pathogenesis, blood-based epigenetic biomarkers have a great promise for early cancer detection. Under specific conditions, those markers can break out of their dormant state and stimulate proliferation, which can ultimately result in a distant relapse and cancer-related death (34). As shown in **Table 4**, different liquid biopsy approaches can be used to detect, characterize, and monitor minimal residual disease in breast cancer, prostate cancer, and melanoma (35).

**Table 4:** Liquid biopsy biomarkers.

Biomarkers	Type of sample	Type of cancer	Method of analysis	References
Fibronectin	Plasma	Breast cancer	ELISA	(32)
Cell free DNA methylation	Plasma, serum	Breast cancer and Prostate cancer	Methylated PCR	(23)
Methylated CYFIP1 gene	cfDNA from Plasma	Sporadic Breast cancer	Genome wide DNA methylation (Illumina methylation assays)	(34)
SPAG6, PER1methylated genes	Cf DNA from plasma	Breast cancer	Pyrosequencing	(34)
ESR1 methylated gene	ctDNA from plasma	Breast cancer	Real time MSP	(34)
DNA methylome	Urine, plasma	Prostate cancer	Methylation Epic Bead-chip (Illumina)	(34)
SNP 8q24 of Myc gene	Plasma	Prostate cancer	Targeted pyrosequencing assays	
Tumor Educated Platelets	Blood	Breast Cancer	mRNA sequencing	(29)

### Extracellular vesicles biomarkers in cancer

**Figure 2:**
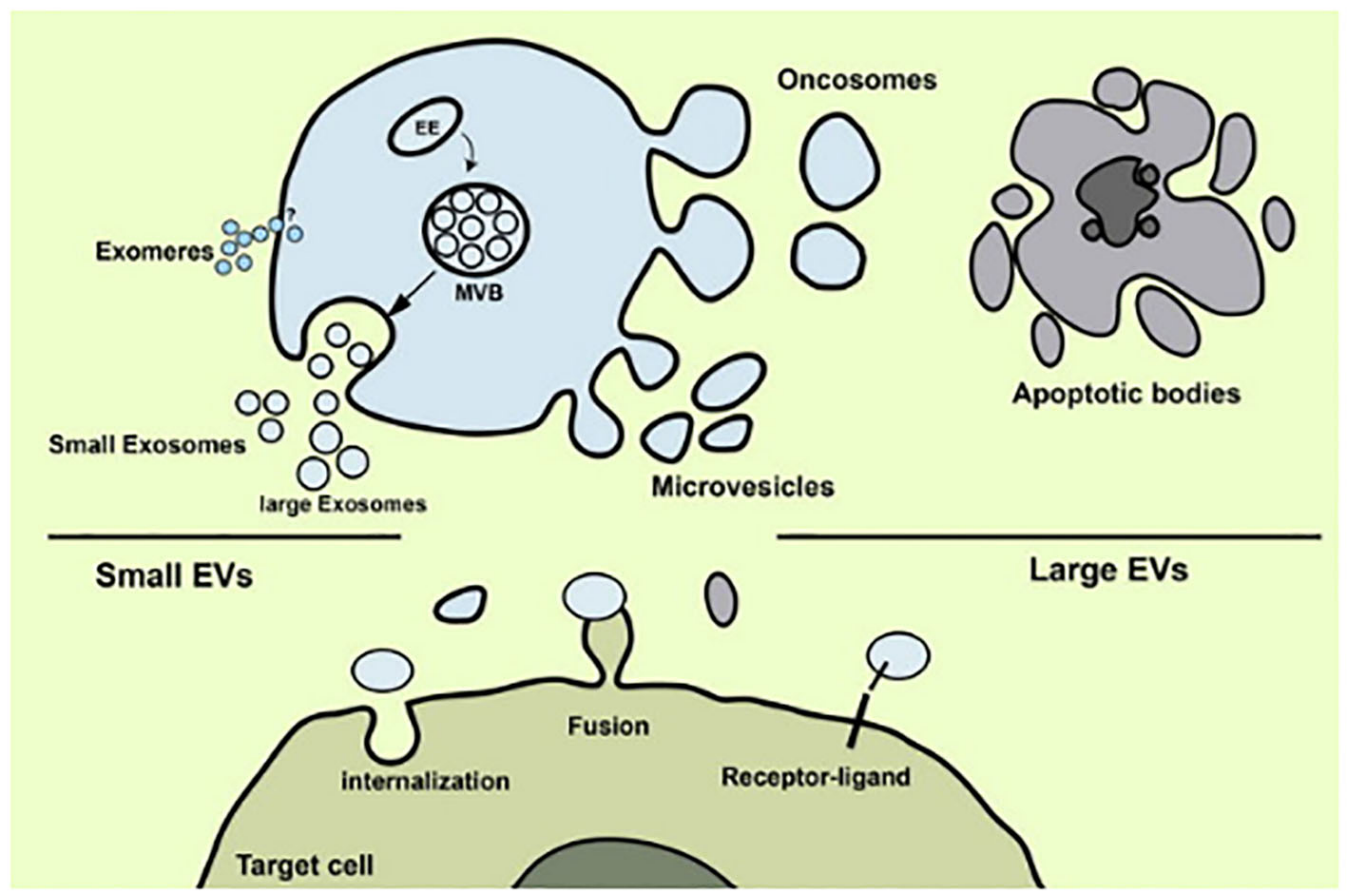
Release of extracellular vesicles of several types (Image inspired from Rezaie et al., 2022) (38).

Extracellular vesicles (EVs) are minute, lipid-bound particles secreted by cells under a variety of diseased and healthy conditions. They transport proteins and nucleic acid as part of their cargo (25). EVs from cancer cells aid in recruiting normal cells to promote tumor growth through proliferative signaling and apoptosis evasion. Research revealed that EVs containing RBM11 from glioblastoma cells induce oncogenic splicing in recipient cells, enhancing survival (36). *In vivo* studies on mice confirmed the malignancy-promoting potential of these EVs (37). Similarly, EVs from glioblastoma cells transfer CLIC1 protein to support tumor growth. Melanoma-derived EVs transfer PDGFR-β, activating PI3K/Akt pathway and inhibiting MAPK pathway in recipient cells, boosting proliferation. Bladder and gastric cancer cell EVs activate PI3K/AKT and MAP/ERK pathways, promoting proliferation and halting apoptosis in recipient cells. Extracellular vesicles may be used as circulating biomarkers for a variety of diseases, including various malignancies, there has been a notable upsurge in scientific interest in these molecules in recent years (**Figure 2**).

Recent years have seen a significant increase in scientific interest in EVs because of their potential use as circulating biomarkers for a wide range of illnesses, including several cancers (39) (see **Table 5**). Studies have shown a significant correlation between the content of EVs, such as proteins, nucleic acids, and lipids, and various aspects of cancer biology, including tumor growth, angiogenesis, immune evasion, and drug resistance (45). The cargo carried by EVs can serve as potential biomarkers for cancer diagnosis, prognosis, and monitoring treatment response.

**Table 5:** EV types that have emerged as biomarkers.

EV type	Cancer type	Biomarker	References
Exosomes	Liver, gastric, breast, colorectal, ovarian, prostate and esophageal cancer.	Diagnosis, therapy tracking, prognosis prediction	(40)
Micro vesicle	Benign and malignant colorectal tumors, bowel cancer.	indicators for dynamic monitoring of cancer progression and prognosis	(41)
Ectosomes	Cancer of the blood	therapeutic targets, progression, and oncogenic transformation	(42)
Proteasomes	Prostate, kidney and bladder	Diagnosis	(43)
Oncosomes	Breast and prostate	Diagnostic/prognostic	(44)
Autophagic EVS	Breast	Breast cancer cells with cytoprotection, management of cancer.	(26)
Apoptotic bodies	Breast and prostate	Therapy	(19)
Exosomes	Liver, gastric, breast, colorectal, ovarian, prostate and esophageal cancer.	Diagnosis, therapy tracking, prognosis prediction	(40)

EV Type: Type of extracellular vesicle analyzed for potential biomarkers in cancer diagnosis and treatment. Cancer Type: Specific types of cancer where these extracellular vesicles are relevant. Biomarker: Indicates whether the EV type is used for diagnosis, therapy tracking, prognosis, or prediction.

*Exosomes*: Serum exosomes from glioblastoma patients contain mutant EGFRvIII mRNA. Zhou and colleagues reported that the exosomal miR-15a-5p expression levels in endometrial cancer are 7–19 times higher than those in other cancer types (46). A poor prognosis was indicated by a substantial correlation between high exosomal miR-1247–3p expression and pulmonary metastases from liver cancer. Since exosomes are released by living cells and may reflect the pathophysiological condition of their parent cells, they are useful indicators for dynamic monitoring of disease progression (26). Increased miR-21 in circulating exosomes has been characterized as a potential biomarker in a number of malignancies, including liver, colorectal, gastric, breast, ovarian, and esophageal cancer. Higher levels of exosomal miR-21 found in urine have been correlated to bladder and prostate cancers (40).

*Microvesicles*: Microvesicles that developed on the cell surface of platelets were demonstrated to discharge lipid-rich molecules having procoagulant potential into their surroundings. The surface shedding, or “ectocytosis,” was later found to occur in a range of cell types, including tumor cells, the cells, neutrophils, and monocyte (47). Both benign as well as malignant colorectal tumor patients had significantly higher plasma concentrations of microvesicles. Microvesicles as biomarkers may improve the usefulness of colon cancer screening systems. Microvesicles are not only useful for detecting cancer, but also serve as biological markers that provide prognoses for many diseases. There are several neurological conditions such as Alzheimer’s disease, epilepsy that have been associated with increases in specific types of circulating microvesicles (48).

*Ectosomes*: Ectosomes are microscopic heterogeneous membrane vesicles that form when several cell types, most typically tumor cells, proliferate from the plasma membrane. They are characterized as a new form of intracellular interaction in which data is sent without physical touch between source and recipient cells. Beyond the plasma membrane, ectosomes are specialized, multipurpose carriers that expand the bounds of a cell. They establish communication networks that let cells share specific traits and information. Ectosomes as potential targets for biomarkers, diagnostic tools, and cancer therapy (41). The state of the living thing from which ectosomes emerge largely determines the specific composition of the substances they convey and their intended purpose. Because tumor-derived ectosomes are present in physiological fluids such as the blood and urine of cancer patients, they may prove to be useful prognostic and predictive biomarkers for breast and prostate cancers. Furthermore, a range of therapeutic modalities may target tumor-derived ectosomes (42).

*Oncosomes*: Extracellular vesicles called oncosomes are excessively large (1–10μm in diameter) and associated with severe disease. They are thought to have originated from malignancy. When membranes bleb shed, they are created. Fluorescence microscopy of large EVs revealed a morphology similar to oncosomes, indicating that these entities are oncosomes (43). One of the proteins localized in oncosomes, cytokeratin 18 (CK18), has been found to be highly prevalent (within the top fifth percentile) and was used in the creation of a test to detect oncosomes in tissues and circulatory of both human and mouse prostate cancer patients. These results imply that oncosomes are a distinct type of extracellular vesicles, or EVs, that can play a variety of roles in the growth of tumors and provide markers specific to malignancy. Potentially useful biomarkers for cancer diagnosis and prognosis are oncosomes. They are applied to prostate and breast cancer (49).

*Prostasomes*: Broadly expressed RNA, membrane, and cytosolic amino acids that are unique to the prostatectomy make up prostatesomes. Extracellular vesicles taken from people with prostate cancer have been shown to include altered levels of protein, mRNA, long non-coding RNA (lncRNA), and microRNA, both in regard to number and quality (27). RNA, which is membrane, and cytosolic amino acids that are unique to the prostatectomy and are broadly expressed make up prostatesomes. The extracellular vesicles taken from people with prostate cancer have been shown to include altered levels of protein, messenger RNA, long non-coding RNA (lncRNA), and microRNA, both in regard to number and quality (28).

**Figure 3:**
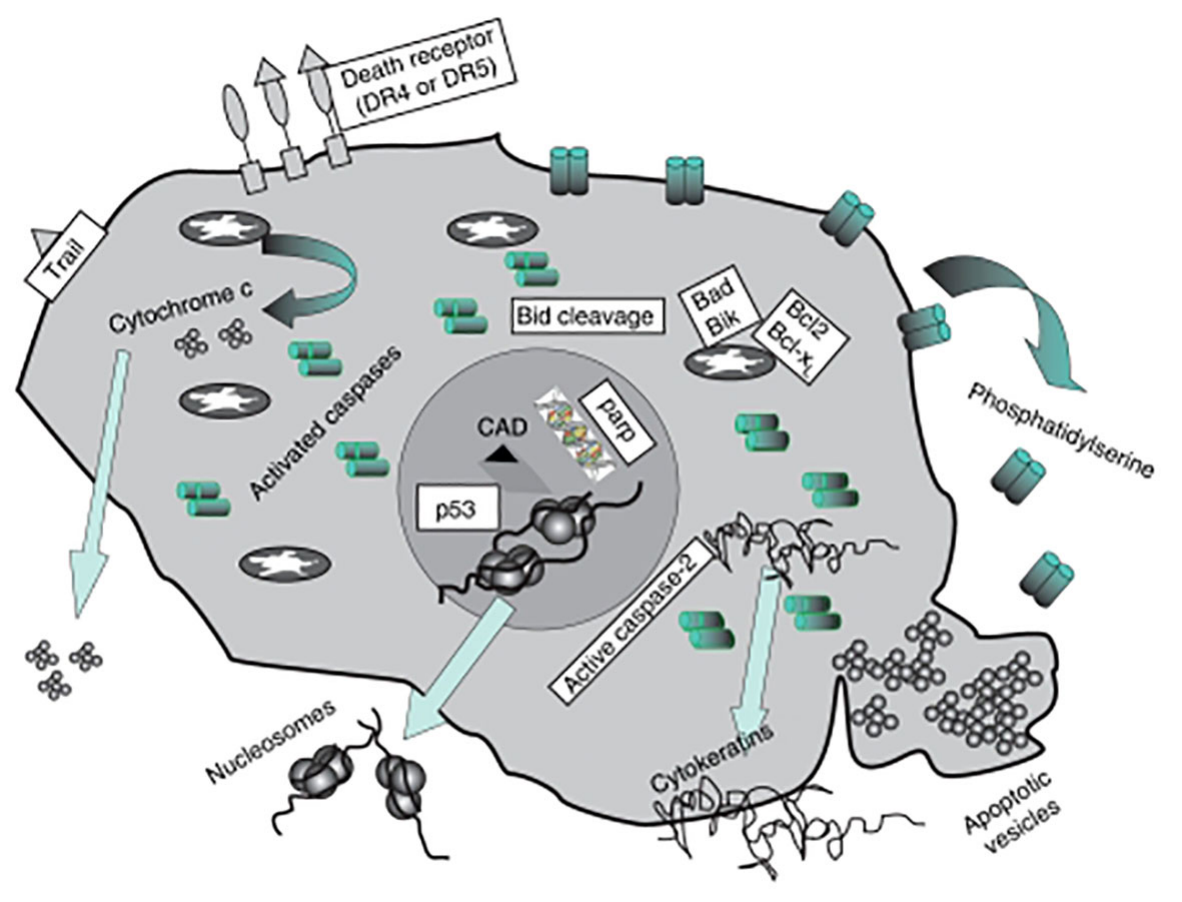
Diagram showing the subsequent protein buildup once apoptosis is induced. Patients receiving medication can eventually release these biomarker molecules into their circulation (Image inspired from Ward et al., 2008) (51).

*Apoptotic bodies*: Cells can perish by a variety of processes, including necrosis, autophagy, and catastrophic mitosis. Currently, however, it is believed that mediators of the death of apoptotic cells have tremendous potential as targets for cancer therapies. Morphological alterations include chromatin and its process of condensation, cell shrinkage, plasma membrane blebbing, and the creation of apoptotic bodies are indicative of apoptosis (50). Cytosolic cytochrome c activates the apoptosome complicated initiating caspase 9, and effector caspases. Apoptotic bodies are produced as a result of a sequence of irreversible events that include the fragmentation of cytokeratins (CKs) by caspase, poly (ADP-ribose) polymerase, with the reactivation of endonucleases to form nucleosomal DNA (nDNA) according to Ye et al, 2020 (**Figure 3**). Furthermore, they promote the exterior of the plasma membrane to become exposed to phosphatidylserine, which allows phagocytes to identify dying cells. Indicators of apoptosis in breast cancer include circulating soluble FASL, granzyme B, and cytochrome C, which increase following treatment. Both intact PCa cells and apoptotic particles made of PCa cells are seen in urine (52). Patients receiving medication can eventually release these biomarker molecules into their circulation as shown in **Figure 2**.

*Autophagic EVS*: Autophagy is one remarkably conserved method of cellular breakdown (53).Cancer cells’ autophagy stimulates the formation of tumors and the division of cancer cells, which inhibits the development of new cancers by killing cancer cells. The autophagic process is regulated by a number of proteins, including class III PtdIns3K complex, Bcl-2, Atg proteins. A degradative organelle known as the vacuole/lysosome receives portions of the cytosol and organelles that are sequestered into an autophagosome, a double-membrane vesicle, for eventual breakdown and recycling of the resultant macromolecules. Thus, autophagy both safeguards breast cancer cells and lessens their sensitivity to medicines. Consequently, autophagy may offer cytoprotection to breast cancer cells (19). Development of protocols to monitor autophagy could be useful in diagnosis and treatment monitoring.

### Epigenetic biomarkers

The elements other than DNA sequence that influence gene expression and cellular phenotypes are referred to as epigenetics (54). The topic of how different phenotypes might be derived from the same genotype is addressed by epigenetics (18). The processes that add acetyl and methyl groups to histone tails, methylate DNA on cytosine residues, express non-coding RNA, and modify the structure of chromatin are the most well-understood epigenetic determinants of phenotype. To maintain the correct differentiation state, cells’ collective epigenetic status is strictly regulated (54–57). This precisely tuned genetic programming is upset in cancer, a process known as epimutation. This results in defective differentiation, unchecked cell division, and resistance to apoptosis (54). A heritable aberrant transcriptional suppression of gene activity that is unrelated to a DNA sequence is known as an epimutation. Although it can also occur in the germline, epimutation usually happens in somatic cells and shows up in the growth of tumors (18). Over the past forty years, epigenetic errors and their causes have become a prominent focus in cancer research when it was discovered that aberrant DNA methylation is associated with malignancy. It has been demonstrated that changes to the epigenome affect almost every stage of the development, growth, and management of tumors (54).

Histone alterations, non-coding DNA, and DNA methylation Since RNAs are found in all human cancer types and can manifest in the early stages of the disease, they make particularly appealing markers with a variety of diagnostic uses (58). Because of their notable stability over RNA and proteins, among other things, DNA methylation and microRNAs are more useful and feasible as biomarkers in therapeutic contexts (59). Specifically, great stability is provided by DNA methylation, microRNAs, and post-translational changes of histones in biofluids and low-quality materials like formalin-fixed paraffin embedded (FFPE) (60). Other advantages of epigenetic biomarkers over genetic or protein-based biomarkers are, that they are dynamic in nature, give more information about the function of the gene, thereby providing information about the specific genetic programs that alter during disease (60). By definition, an epigenetic biomarker is “any altered epigenetic mechanism or mark that is specifically stable and reproducible during sample processing and is generally used to evaluate health or disease status” (60).

### DNA methylation in cancer

The most extensively researched epigenetic modification in cancer is aberrant DNA methylation (61). In eukaryotic cells, aberrant hypermethylation of promoters can silence critical genes, including tumor suppressor genes, which in turn can cause illness. The reverse process can also have an impact on the development of cancer. Genes that are typically methylated, such as oncogenes, can have their expression elevated by hypomethylation (62). It is interesting to note that the first DNA methylation anomaly in human cancer to be discovered was hypomethylation (63). It has been found that 13% of sporadic colorectal cancer (CRC) show MLH1 hypermethylation, and a BRAF c.1799T>A, p.Val600Glu mutation has often also been identified in tumor DNA (64, 65). Though it is brought on by mutations in one of the DNA MMR genes, MSI and loss of MLS1 are both present in Lynch syndrome, the most frequent cause of hereditary colorectal cancer (66). It has been discovered that methylation of MGMT occurs in 40% of tumors in gliomas and CRC, but only in 25% of tumors in non-small cell lung carcinomas (NSCLCs), lymphomas, and head and neck carcinomas (67). When paired with IDH1 mutations, MGMT methylation status functions as a predictive biomarker. Patients with hypermethylated MGMT and the IDH1 p.R132H mutation had a better prognosis for their glioma (68). MGMT is a DNA repair gene that helps to eliminate harmful and mutagenic alkyl groups from O6-meG. Due to the fact that DNA alkylation causes mutations, MGMT shields cells from harm (67, 69).

The primary association of the RB1 gene with retinoblastoma is the loss of RB1 function. LOH or RB1 mutations are linked to the lack of expression of this gene in retinoblastoma and other malignancies, such as bladder carcinomas and malignant neuroendocrine lung carcinomas. However, methylation of RB1 might sometimes result in the suppression of its expression (70, 71). It has been said that RB1 methylation above the LOH and mutations are required for complete molecular diagnoses of retinoblastoma. It has been reported that 9% of spontaneous unilateral tumors are caused by RB1 hypermethylation, which is invariably acquired (72).

Circulating methylation SEPT9 DNA is one type of plasma epigenetic biomarker for colorectal cancer screening. SEPT9 is regarded as a tumor suppressor because it controls cell proliferation and inhibits unchecked cell division (73). Research has shown that SEPT9 methylation is linked to the pathophysiology of colorectal cancer (CRC), and that a decline in SEPT9 expression is connected with the advancement of neoplastic illness (74). SHOX2 hypermethylation has been noticed in the bronchial aspirates [43], pleural effusions [44], and blood plasma of patients with lung cancer (18, 75). DNA methylation analysis of SHOX2 combined with PTGER4 in blood plasma allows detection of lung cancer and differentiation of non-malignant diseases (75). In some cancer there are some epigenetic markers that has been discovered due to the fact that prostate cancers frequently contain methylation of the tumor suppressor genes GSTP1, RASSF1, and APC, these genes are regarded as cancer biomarkers (**Table 6**) (18).

**Table 6:** Methylation as prognostic and predictive biomarkers.

Methylation	Diagnostic method	Cancer type	References
MLH1 Hypermethylation	Invasive	Colorectal cancer	(76)
MGMT Hypermethylation	Invasive	Glioblastoma	(77)
IDH1 p.R132H mutation and MGMT hypermethylation	Invasive	Glioblastoma	(78)
RB1 hypermethylation	Invasive	Retinoblastoma	(79)
GSTP1, RASSF1, APC methylation status	Invasive	Prostate cancer	(80)
SEPT9	Non-Invasive	Colorectal cancer, Lung cancer	(81, 82)
ZNF331	Invasive	Colorectal cancer	(83)
MGMT-STP27	Invasive	Oligodendrogliomas and oligoastrocytomas	(84, 85)
ESR1	Non-Invasive	Breast cancer	(86)
MGMT Hypermethylation	Invasive	Glioblastoma	(87)
SALL1	Invasive	Head and neck cancer	(88, 89)

Footnotes: Methylation: Refers to the methylation status of specific genes which can serve as biomarkers. Diagnostic Method: The approach used to analyze the methylation status. Cancer Type: Types of cancers where these methylation markers are relevant. Prognostic Biomarkers: Markers used to predict the overall outcome or course of the disease. Predictive Biomarkers: Markers used to predict the likely response to treatment.

### Histone Modification in Cancer

Generally, chromatin can be divided into two categories: euchromatin, which is more loose and contains actively transcribed genes, and heterochromatin, which is heavily compacted and contains dormant genes (18). The arrangement and functionality of chromatin are changed by covalent alteration of the histones that make up nucleosomes, which has an impact on the regulation and expression of genes (90). The six main roles of chromatin are transcription, repression, enhancer, insulator, promoter, and inactive chromatin. Histone modification is a significant factor in determining the function of chromatin (91). Phosphorylation, acetylation, methylation (mostly of lysine and arginine residues), ubiquitylation, glycosylation, SUMOylation, ADP (adenosine diphosphate)-ribosylation, and carbonylation are examples of modifications to histone structure (90, 92). The primary indicators of active chromatin are histone acetylation and methylation, which are frequently linked to a more relaxed chromatin conformation. Conversely, chromatin condensation is frequently linked to histone deacetylation and phosphorylation, which are indicators of inactive chromatin (90).

Globally methylated or non-modified histones were linked to a poor prognosis, whereas individuals with NSCLC who had global histone acetylation had a better prognosis in survival analysis (93). Human tumor cells are typically characterized by a complete loss of H4 histone Lys16 monoacetylation and Lys20 trimethylation, which is linked to DNA hypomethylation. A lower level of H4Lys20 methylation and H4Lys16 acetylation in breast cancer is associated with a worse prognosis (94, 95). The propensity for cancer can be increased by different isoforms of histone proteins found in the nucleosome as well as covalent alterations. For instance, genitourinary malignancies, which include bladder and prostate tumors, and undifferentiated cancers have been shown to overexpress the H2A histone isoform H2A.Z. Moreover, H2A.Z may contribute to endocrine resistance in individuals with breast cancer. The association between H2A.Z levels and short overall patient survival suggests that H2A.Z may be a valuable biomarker for tumor progression (96). In terms of post-translational histone changes, CRC patients’ blood had lower levels of H3K9me3 and H4K20me3 than that of cancer-free people (97). Global patterns of histone H3 and H4 modification are significant because they may serve as indicators of both disease-free survival and tumor recurrence (**Table 7**).

**Table 7:** Histone modification markers.

Histone modifications	Method of diagnostics	Source	Cancer type	References
H3Cit	Non-invasive	Blood	Advanced cancers	(98)
cf-nucleosome epitope combination	Non-invasive	Blood	Colorectal cancer	(99)
H3K4me3 and Wdr82 expression	Non-invasive	Blood	Colorectal cancer	(100)

Footnotes: Histone Modifications: Specific modifications to histones analyzed as potential biomarkers. Method of Diagnostics: Techniques used to detect histone modifications. Source: Biological source from which the samples are taken for analysis. Cancer Type: Types of cancers where these histone modifications are relevant.

### MicroRNA (miRNA) in Cancer

The second most extensively researched epigenetic method of gene regulation, after methylation, is the interaction between microRNA (miRNA) and messenger RNA (mRNA). These are 18–25 nucleotide short, non-coding RNA molecules that are essential for controlling post-transcriptional gene expression. Translation is halted by miRNAs when they bind to the target mRNA molecule. It is believed that miRNA can regulate up to 60% of genes that encode proteins (18). Saliva, urine, serum, and plasma are among the bodily fluids into which tumor cells release miRNAs. Thus, the examination of circulating miRNAs in liquid biopsy samples offers potential biomarkers for non-invasive diagnostics in numerous human cancers, such as melanoma and rhabdomyosarcoma, as well as colorectal, lung, breast, prostate, gastric, pancreatic, esophageal, liver, thyroid, kidney, ovarian, endometrial, and cervical cancers (101). The control of numerous genes implicated in the genesis of cancer is greatly impacted by dysregulation of miRNA expression (18). The suppressor gene may be silenced as a result of overexpression of miRNA, which is implicated in the negative regulation of the suppressor gene. Conversely, overexpression of the oncogene occurs when the chromosomal loci encoding the miRNA that silences the oncogene are deleted. Therefore, oncogenes (oncomiRs) and miRNAs themselves can function as suppressors. It is also critical to keep in mind, that while a single miRNA molecule can control several genes, multiple miRNAs can target a single mRNA (18). Since miRNA activity and expression changes along the course of cancer development, miRNAs can function as biomarkers and be assessed in cancer patient blood and tumor tissue (**Table 8**) (18).

**Table 8:** miRNA markers for cancer.

MiRNA	Prognostic	Predictive	Invasive/non-invasive diagnostic	Cancer type	Biological sample	References
miR-21	+	+	Invasive/non-invasive	Multiple types of cancers	Blood/tissue	(102)
miR-30d, miR-21	+	–	Invasive	Non-small cell lung cancer	Tissue	(103)
miR-31–3p	+	+	Invasive	Colorectal cancer	Blood/tissue	(104)
miR-106a, miR125a-5p, miR129–3p, miR-205, miR-21, miR-29b, miR-375, miR-7	+		Invasive	Non-small cell lung cancer	Tissue	
miR-29a, miR-92a	+	–	Non-invasive	Colorectal cancer	Blood	(105)
miR-506, miR-4316	+	–	Non-invasive	Colorectal cancer	Blood	(106, 107)
miR-126, miR-145, miR-210, miR-205–5p	+	–	Non-invasive	Non-small cell lung cancer	Blood	(108)
miR-149–3p, miR-150–5p, miR193a-3p	+	–	Non-invasive	Melanoma	Blood	(109)
miR-200 family	–	+	Non-invasive	Ovarian,	Blood	(110)
miR-17, miR-17–5p	+	+	Non-invasive, invasive	Breast cancer, gastric cancer	Blood, tissue	(111, 112)
miR-155	–	+	Invasive	Lung cancer	Tissue, serum/plasma	(113)

Footnotes: miRNA: MicroRNAs analyzed as potential biomarkers. Prognostic: Indicates whether the miRNA is used for prognostic purposes. Predictive: Indicates whether the miRNA is used for predictive purposes. Invasive/Non-invasive Diagnostic: Indicates whether the miRNA can be detected through invasive or non-invasive methods. Cancer Type: Types of cancers where these miRNA markers are relevant. Biological Sample: The type of sample (e.g., blood, tissue) used for analysis.

The combination of two miRNA biogenesis genes (DICER1 and DROSHA) and four miRNAs (miR-30d, miR-21, miR-17, and miR-155) is one of the putative predictive indicators in non-small cell lung cancer (114). Specifically, miR-30d has been discovered to function as an oncomiR in cancer, and a substantial decrease in lifespan is associated with an increased copy number of miR-30d in cancer tissue (gains or amplifications compared to others) (114, 115). miR-31–3p is an additional intriguing prognostic and predictive biomarker in metastatic colorectal cancer (116). Blood alterations are a reflection of the dysregulation of many miRNAs found in malignancies. Let-7a-1, 7a-2, 7a-3, 7b, 7c, 7d, 7e, 7f-1, 7f-2, 7g, 7i, miR-98, and miR-202 are a group of well-known miRNAs that have changed expression in malignancies (117). Since some members of the let-7 family are downregulated in melanoma, pancreatic cancer, prostate cancer, and sarcoma, the let-7 family is thought to be a tumor suppressor (118). However, they can also be upregulated in lymphoma, mesothelioma, and breast cancer (118). It was discovered that miR-21 acts as an oncogene by suppressing the expression of many tumor suppressors (119). For instance, the miRNA targets PDCD4, which is linked to the prevention of neoplastic transformation as well as the promotion, invasion, and advancement of cancer (118–120). Among the other targets of miR21 are BCL2, PTEN, RECK, RHOB, and TPM1 (121). This miRNA has been linked to hematological malignancies, breast cancer, gastric cancer, ovarian cancer, pancreatic cancer, colorectal cancer, lung cancer, and liver cancer in terms of its diagnostic, predictive, and/or prognostic properties (18).

### Non-coding RNA biomarkers

**Figure 4:**
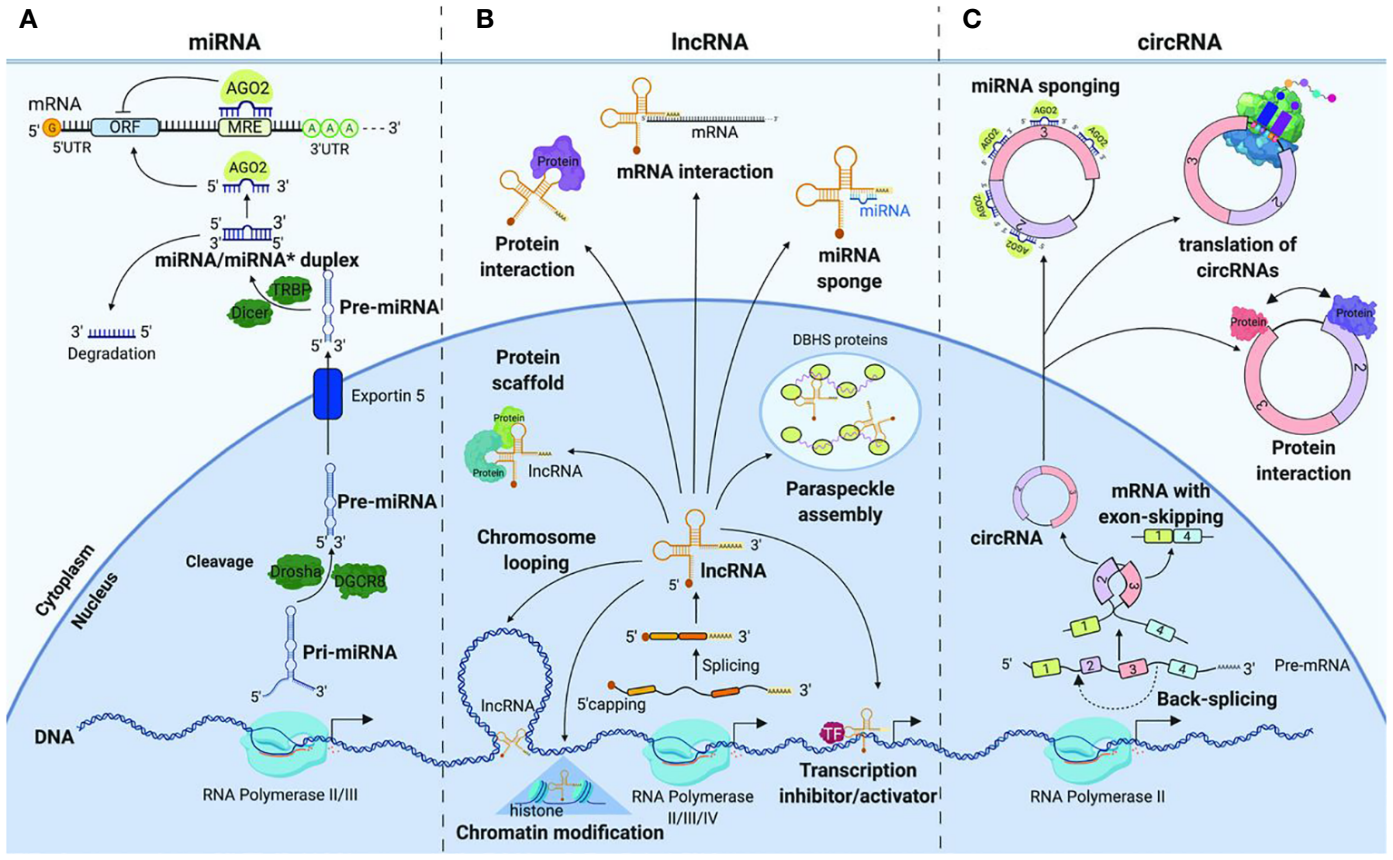
Non-coding RNAs in gastrointestinal cancer (Image inspired from Dragomir et al., 2019) (124).

In recent years, extensive research has elucidated the multifaceted roles of ncRNAs in cancer. Non-coding RNAs (ncRNAs) have emerged as pivotal players in the intricate landscape of cancer biology, contributing significantly to both the understanding of malignancies and the development of innovative therapeutic strategies (122, 123). These molecules, including microRNAs (miRNAs), long non-coding RNAs (lncRNAs), and circular RNAs (circRNAs), exert regulatory functions in gene expression and cellular processes (**Figure 4**). The dysregulation of ncRNAs has been linked to various malignancies, making them attractive candidates for diagnostic and therapeutic applications.

*MiRNAs*: MiRNAs, small non-coding RNA molecules, have garnered attention for their involvement in cancer pathogenesis (125–129). Notably, elevated levels of Phosphatase and Tensin Homolog (PTEN) induced by specific miRNAs can inhibit AKT signaling, activate apoptosis, and prevent malignancies such as renal cell carcinoma (130, 131). MiRNAs, including miR-29a, have demonstrated a major impact on oncogenicity in various neoplasms by regulating key genes involved in cancer progression (128). These molecules play crucial roles in physiological and pathological processes, including viral replication, cell proliferation, differentiation, apoptosis, fibrosis, angiogenesis, tumorigenicity, metastasis, and drug resistance. Research has identified specific miRNA panels with distinct expression patterns in the serum of cancer patients, offering potential as diagnostic indicators (132). For instance, a combination of miR-145, miR-155, and miR-382 demonstrated improved sensitivity and specificity, suggesting the potential of miRNA profiling for breast cancer screening (132). The translational applications of miRNAs extend to the management and survival improvement of oral cancer patients (129).

*LncRNAs*: Sometimes referred to as versatile biomarkers across cancers, long non-coding RNAs (lncRNAs) have emerged as versatile biomarkers with diagnostic potential across various cancers (133). A systematic review and meta-analysis highlighted lncRNA AFAP1-AS1 as a novel biomarker in different cancers (134). Specific lncRNAs, such as MALAT-1, HOTAIR, LINC00152, and others, have shown diagnostic potential in prostate, lung, colorectal, hepatocellular, gastric, renal, and colorectal cancers (135). LncRNA GIHCG has been linked to the etiology of numerous malignancies, offering promise as a biomarker (136). Additionally, circulating lncRNAs, like PVT1 and UCA1, exhibit significant multicancer diagnostic potential (137). These findings underscore the diversity of lncRNAs as biomarkers and their potential application in clinical setting.

*CircRNAs*: Circular RNAs (circRNAs) represent a novel class of non-coding RNAs with emerging roles as biomarkers and therapeutic targets in cancer (138). The discovery of their involvement in the onset and progression of malignancies has opened new frontiers in cancer research. CircRNAs such as circ0001955 and circ-LDLRAD3 have shown promise as diagnostic and prognostic markers in cervical and pancreatic cancers, respectively (139). Notably, exosomal circ_0044516 was found to be highly elevated in prostate cancer patients, influencing cancer cell proliferation and metastasis by modulating miR-29a-3p expression (140). These findings underscore the potential of circRNAs as valuable biomarkers and therapeutic targets in diverse cancer types.

### Metabolomic biomarkers in cancer

Metabolomic biomarkers refer to specific small molecule metabolites produced by the organism in biological fluids that can be identified and analyzed to provide insights into physiological or pathological states. These biomarkers, detected through nontargeted metabolomic analysis, serve as indicators of metabolic changes associated with various conditions, including diseases or responses to treatments. Essentially, metabolomic biomarkers are measurable metabolic features that can be used for diagnostic, prognostic, or therapeutic purposes, providing valuable information about the biochemical status of an organism (141). These compounds typically weigh ≤ 1500 Da and span a diverse range, including peptides, oligonucleotides, sugars, nucleosides, organic acids, ketones, aldehydes, amines, amino acids, lipids, steroids, alkaloids, and occasionally drugs or xenobiotics (141). Metabolomic biomarkers have emerged as promising tools for non-invasive diagnostic and treatment monitoring in oncology. Metabolomics technologies have advanced our understanding of cancer metabolism, particularly in the context of the “Warburg effect,” which elucidates how cancer cells utilize glycolysis to support tumor proliferation and vascularization (142, 143).

**Figure 5:**
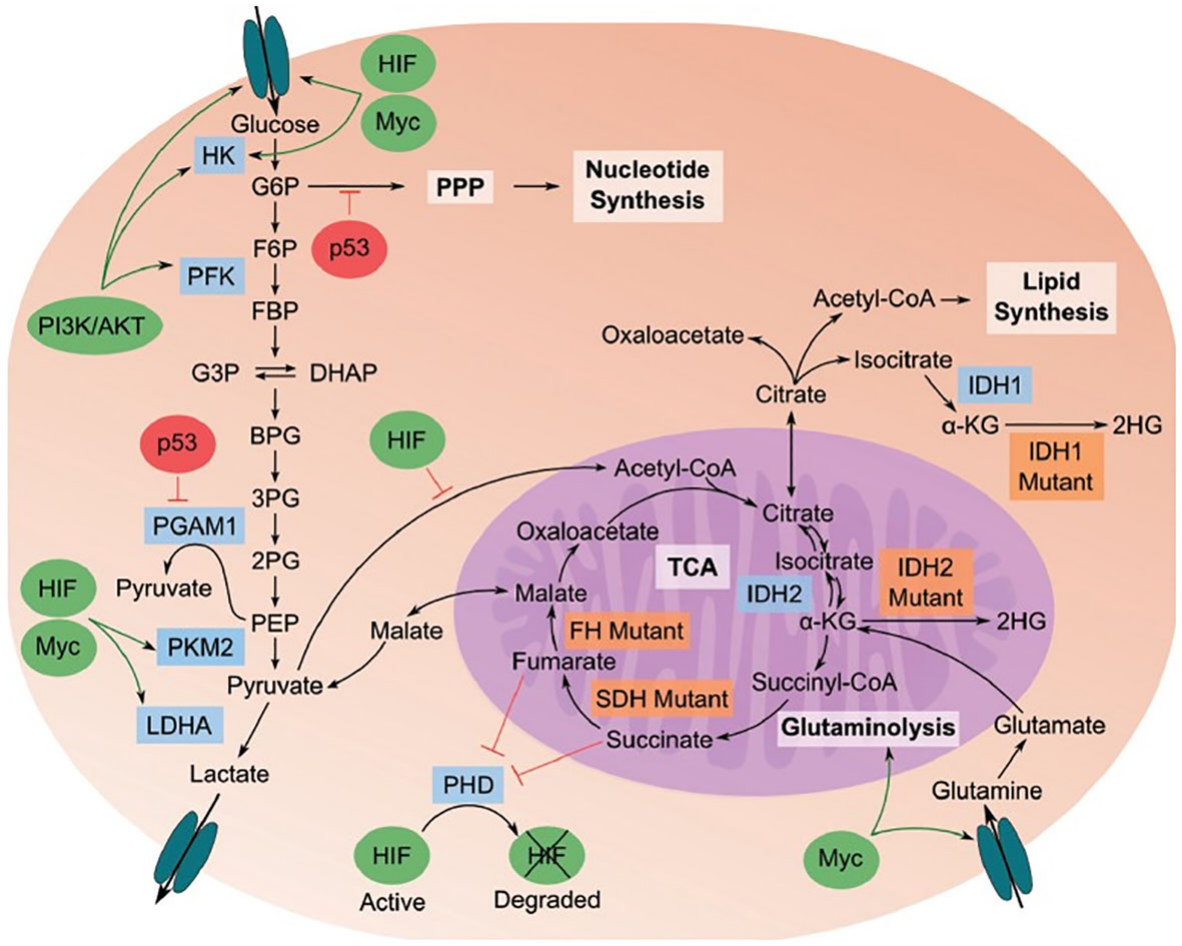
Cancer metabolome showing the relationships between metabolome, proteome, and genome in cancerous cells (Image inspired from Bhattacharjee et al., 2022) (144).

The tumor metabolome provides insights into the interconnectedness of metabolome, proteome, and genome within cancer cells. Cancer cells exhibit altered metabolic processes, such as glycolysis, which converts glucose into pyruvate and subsequently ferments it into lactate (**Figure 5**).

The flow of pyruvate through the TCA cycle is reduced in cancer cells. Additionally, pathways stemming from glycolysis, like the pentose phosphate pathway, generate essential building blocks to support the rapid growth of cancer cells. Specific genetic and enzyme-related behaviors play a role in this process. Enzymes highlighted in blue are crucial for the transition to a cancer metabolic phenotype, while those in orange indicate mutations found in cancer cells. Oncogenes, represented by green ovals, are up-regulated in cancer, whereas tumor suppressors, depicted by red ovals, are down-regulated.

In breast cancer, the integration of genomics, proteomics, and metabolomics has been proposed as a key approach for future biomarker discovery, highlighting the potential of metabolomics in this field (145). Furthermore, computational models applied to metabolomics data have hinted at the relevance of glutamine metabolism in breast cancer, emphasizing the potential of metabolomics in the development of new biomarkers for cancer (146). Metabolomics holds great promise for understanding the molecular determinants of cancer and advancing the development of new biomarkers for the diagnosis, prognosis, and treatment of neoplastic processes (147). Additionally, metabolomics offers a broad set of oncological applications, particularly in providing serum or imaging-based biomarkers for cancer (148). The utility of metabolomics in biomarker discovery for cancer has been demonstrated in various types of cancer, including colorectal cancer, where it has proven useful in early diagnostic biomarker discovery (149). The potential of metabolomics in cancer research extends to its application in precision medicine, as it can suggest new pathways and therapeutic targets for the targeted treatment of cancer (150).

Recent advances in metabolomics technologies have enabled a deeper investigation into cancer metabolism, providing a better understanding of how cancer cells utilize metabolic pathways for proliferation and vascularization (142), for instance, metabolic biomarkers for breast cancer, with a focus on glutamine metabolism (146). Additionally, metabolomics has been applied to urine and saliva for non-invasive cancer detection and biomarker discovery (151). Furthermore, metabolomics represents a potential strategy for the real-time selection and monitoring of patients treated with immunotherapy, indicating its relevance in treatment monitoring in oncology (152). The integration of metabolomic profiling with transcriptomics data has been proposed as a method for validating potential diagnostic biomarkers in cervical cancer, further emphasizing the potential of metabolomics in cancer research (153). Recent studies have shown the potential of metabolomics in identifying biomarkers for various cancers, such as endometrial cancer and primary glomerulonephritis sub-types (154) (**Table 9**).

**Table 9:** Some metabolomic markers used as non-invasive markers along with biological samples.

Cancer type	Metabolomic markers	Biological sample	References
Prostate	Alanine, Arginine, Uracil, Glutamate, Fumarate, Citrate	Tissue, Urine, Blood Plasma/Serum, Prostatic Fluid, Immortalized Cultured Cell Lines, Extracellular Vesicles (EVs) from Urine	(155) (156) (157)
Breast	Hypotaurine, Pathway-based metabolomic features	Tissue, Plasma, Urine, Extracellular Vesicles (EVs) from Urine, Nails, Plasma	(158) (159) (160)
Kidney	Circulating tumor cells, Circulating RNAs, Cell-free proteins, Exosomes	Blood, Urine	(161)
Lung	Exosomal miRNAs, Metabolites	Sputum, Exhaled breath condensate, Blood, Urine	(162) (163)
Colorectal	Fecal nucleatum, Microbial markers,	Feces, Blood, Tissue	(164)
Blader Cancer	benzoic acid, hippuric acid, and 4-hydroxycinnamic acid	Urine	(165)

## Uninvited biomarkers in cancer genomics research in sub-saharan africa

Cancer care in sub-Saharan Africa is challenged by several unique issues that contribute to worse outcomes compared to high-income countries (1). Most patients present with metastatic stage disease due to delayed diagnosis, secondary to low cancer awareness among both the population and healthcare workers. There are also cultural and economic barriers that hinder access to specialized care. Paradoxically, African populations are among the least studied in cancer genomics globally. Even though Africa is the most genetically diverse continent, it makes up only about 3% of the genetic data used in cancer genomics projects worldwide (2). Consequently, the genetic determinants of cancer risk and treatment response in African populations remain largely unknown. The limited cancer genomics research in Africa is also unevenly distributed, with studies primarily focused on North African populations, while sub-Saharan Africa remains vastly unexplored (3). This is problematic, as the genetic underpinnings of cancer can differ greatly across African subpopulations due to the continent’s immense genetic diversity. Another key issue is the tendency to treat Africans as a homogeneous group in cancer research, rather than disaggregating by ancestry, ethnicity or language (4). This obscures important within-group differences in cancer risk and biology, hindering the achievement of true equity in precision oncology.

Despite these challenges, recent cancer genomics studies in Africa have uncovered several “uninvited biomarkers” with potential clinical utility (5). For instance, inflammatory markers like C-reactive protein and certain cytokines have been linked to prognosis and cancer stage in African cohorts. Metabolic profiling has also revealed distinct patterns associated with common malignancies in the region. Notably, viral integrations, particularly from hepatitis B virus and human papillomavirus, have emerged as an intriguing category of biomarkers (6). These viral sequences integrated into the host genome can dysregulate critical cellular pathways, impacting cancer biology and behavior. Importantly, the foreign viral antigens expressed by these cancers offer opportunities for targeted therapies and immunotherapeutic strategies. Addressing the unique challenges in cancer care and genomics research in sub-Saharan Africa is crucial to improving outcomes and achieving equity in precision oncology. Sustained funding, multidisciplinary collaboration, and empowerment of African scientists are essential to drive progress in this field and ultimately reduce the devastating cancer burden in the region.

## Conclusions

Non-invasive biomarkers, including liquid biopsies, epigenetic markers, non-coding RNAs, exosomal cargo, and metabolites, have emerged as promising tools in cancer diagnosis and treatment. The systematic review provides a comprehensive overview of the potential of these biomarkers in early detection, disease monitoring, and personalized treatment strategies across various cancer types. The ability to accurately detect cancer in its early stages and classify subtypes have significant implications for improving patient outcomes and advancing oncology. The studies reviewed in this article demonstrate the ability of non-invasive biomarkers such as liquid biopsies, epigenetic markers, non-coding RNAs, exosomal cargo, and metabolites to accurately detect cancer in its early stages, classify subtypes, and personalize treatment regimens. Understanding the roles of ncRNAs in cancer, not only provides insights into the intricate molecular mechanisms driving malignancies, but also paves the way for the development of targeted therapeutic interventions. Sorafenib, a multi-kinase inhibitor, stands out as an example of the successful translation of ncRNA research into cancer therapy, having been approved by U.S. Food and Drug Administration for the treatment of advanced renal cell carcinoma, hepatocellular carcinoma, and thyroid cancers. The development of these biomarkers represents a significant advancement in oncology, offering new avenues for improving patient outcomes and reducing the burden of cancer worldwide. Continued research and validation are necessary to establish these biomarkers as reliable tools in clinical practice, thereby, ultimately contributing to the development of more effective cancer management strategies.

### Further research and potential future directions

While the studies reviewed in this article provide compelling evidence for the utility of non-invasive biomarkers in cancer medicine, there is still much work to be done in this field. Despite the advances in cancer biomarker research, several limitations hinder their clinical application. One significant issue is the lack of standardized protocols for biomarker detection and quantification, leading to variability in results across different studies and clinical settings (166). Additionally, many biomarkers currently lack sufficient validation in large, diverse patient populations, which raises concerns about their generalizability and reliability (167). Future research should focus on validating these biomarkers in larger patient cohorts, exploring their potential for use in combination with existing diagnostic and treatment modalities, and developing new technologies to enhance their sensitivity and specificity. Another limitation is the complexity of cancer biology, which makes it challenging to identify biomarkers that are both highly specific and sensitive for early detection and prognosis. Current biomarkers often fail to capture the heterogeneity of cancer, leading to false positives and negatives (168). There is need for more extensive research into the underlying mechanisms of the origin of these biomarkers and their significance in cancer biology. This knowledge will be crucial for developing more effective therapies that target the specific molecular pathways involved in cancer development and progression. Early detection is very important for the improvement of life quality, survival, and to reduce the financial burden of cancer treatments, which are greater at later stage detection. Moreover, the integration of biomarkers into clinical practice is complicated by the lack of robust bioinformatic tools to analyze and interpret large-scale biomarker data (169). To address these challenges, future research should focus on the following areas:

*Standardization and Validation*: Develop standardized protocols for biomarker detection and validation through multi-center studies involving diverse patient populations. This will help ensure the reliability and reproducibility of biomarker tests across different settings (166).

*Multi-omics Approaches*: Integrate genomic, transcriptomic, proteomic, and metabolomic data to identify composite biomarker signatures that better reflect the complexity of cancer. This holistic approach can improve the specificity and sensitivity of biomarkers (167).

*Advanced Bioinformatics*: Invest in the development of advanced bioinformatic tools and machine learning algorithms to analyze complex biomarker data. These tools can help uncover novel biomarker patterns and improve the interpretation of existing data (169).

*Ethical and Safe Integration of AI*: Explore the ethical and safe integration of artificial intelligence (AI) in cancer research to enhance biomarker discovery and application. AI can help in the rapid analysis of large datasets and the identification of potential biomarkers with high clinical relevance (170).

*Combining Biomarkers with Existing Therapies*: Investigate the potential of combining biomarkers with existing therapeutic strategies to enhance treatment efficacy and overcome resistance. This includes exploring the role of biomarkers in predicting and monitoring treatment response (171).

## Author contributions

SZ: Conceptualization, Methodology, Writing – original draft, Writing – review & editing. NKN: Validation, Writing – review & editing. GVO: Conceptualization, Writing – original draft. OO: Visualization, Writing – review & editing. PCN: Resources, Writing – original draft. MT: Data curation, Writing – original draft. ENI: Investigation, Writing – original draft. SEA: Project administration, Writing – review & editing. OOO: Project administration, Writing – review & editing, Supervision. 
